# Large tumour volume reduction of IDH-mutated anaplastic glioma involving the insular region following radiotherapy

**DOI:** 10.1186/s12883-021-02548-3

**Published:** 2022-01-13

**Authors:** Gabrielle Metz, Dasantha Jayamanne, Helen Wheeler, Matthew Wong, Raymond Cook, Nicholas Little, Jonathon Parkinson, Marina Kastelan, Chris Brown, Michael Back

**Affiliations:** 1 Department of Radiation Oncology, Northern Sydney Cancer Centre, Royal North Shore Hospital, St Leonards, Sydney, NSW 2065 Australia; 2 Sydney Medical School, University of Sydney, Sydney, Australia; 3 Genesis Cancer Care, Sydney, Australia; 4 The Brain Cancer Group, Sydney, Australia; 5 Central Coast Cancer Centre, Gosford Hospital, Gosford, Australia; 6 Department of Neurosurgery, Royal North Shore Hospital, Sydney, Australia

**Keywords:** Anaplastic glioma, IDH mutation, Residual volume, Radiation therapy

## Abstract

**Background:**

The impact of near-total resection of IDH-mutated anaplastic glioma (IDHmutAG) is well-established but there remains uncertainty of benefit in tumours of the insular cortex where the extent of safe resection may be limited. This study aimed to assess tumour volume reduction in patients following IMRT and impact of residual post-surgical volume.

**Methods and materials:**

Patients with IDHmutAG involving insular cortex managed with IMRT from 2008 to 2019 had baseline patient, tumour and treatment factors recorded. Volumetric assessment of residual disease on MRI was performed at baseline, month+ 3 and month+ 12 post-IMRT. Potential prognostic factors were analysed for tumour reduction and relapse-free survival, and assessed by log-rank and Cox regression analyses.

**Results:**

Thirty two patients with IDHmutAG of the insular cortex were managed with median follow-up post-IMRT of 67.2 months. Pathology was anaplastic astrocytoma (AAmut) in 20, and anaplastic oligodendroglioma (AOD) in 12 patients. Median pre-IMRT volume on T1 and T2Flair was 24.3cm^3^ and 52.2cm^3^. Twenty-seven patients were alive with 5-year relapse-free survival of 80%. There was a median 67 and 64% reduction from baseline occurring at 3 months post-IMRT for T1 and T2Flair respectively; and subsequent median 78 and 73% at 12 months. At 12 months AOD patients had median 83% T1 volume reduction compared to 63% in AAmut (*p* < 0.01). There was no difference on T2Flair volume (*p* = 0.64). No other pathological factors influenced volume reduction at 12 months. No factors were associated with relapse-free survival including baseline T1 (*p* = 0.52) and T2Flair (*p* = 0.93) volume.

**Conclusion:**

IMRT provides large tumour volume reduction in IDHmutAG of the insular cortex. While maximal safe debulking remains standard of care when feasible, this patient cohort reported no significant negative impact of residual disease volume on relapse-free survival.

## Introduction

Anaplastic gliomas (AG) with IDH mutation (IDHmut) involving the insular cortex of the frontal lobe are infiltrative tumours that extend diffusely along neural tract pathways. These tumours occur in younger patients and have median survivals beyond 10–15 years [1–4]. Improved surgical techniques allow an attempt for complete resection [5, 6], however due to the anatomical site there is potential risk of long-term post-surgical neurological deficits [7]. Whilst evidence supports an association of complete surgical resection and improved outcome for glioblastoma and IDH wildtype anaplastic glioma [8, 9], there is conflicting evidence for similar magnitude of benefit for IDH mutated anaplastic glioma [10, 11]. The landmark data from Berger et al. demonstrates an association with surgical volume reduction and outcome, however, this was in the era prior to IDH diagnostic classification and additionally 40% of patients received radiation therapy following surgery [12].

Concurrently improvements in radiation therapy techniques and target volume delineation of tumour can minimise risk of morbidity and potentially provide a greater therapeutic ratio with reduction of risk [13–15]. This study explores the volume reduction, morbidity and survival occurring in patients with AG IDHmut involving the insular cortex managed with IMRT and adjuvant temozolomide.

## Methods

Consecutive adult patients diagnosed with anaplastic glioma and referred to the Neuro-oncology Multidisciplinary Tumour Board at the Northern Sydney Cancer Centre were entered into a prospective Anaplastic Glioma Database, approved by The Institutional Ethics Review Board. All methods were performed in accordance with relevant guidelines and regulations and all patients provided informed consent via an opt-out policy [16].

### Patient selection

Eligible patients for this study were those patients in the Anaplastic Glioma Database with an IDH-mutated tumour with extension into the insular region, and managed with IMRT between January 2008 and June 2019. In the database, histopathological classification was initially recorded as anaplastic astrocytoma (AA), anaplastic oligoastrocytoma (AOA) or anaplastic oligodendroglioma (AOD) based on WHO 2007 Classification [16, 17]. Molecular factors such as 1p19q codeletion, IDH1/2 mutation (evaluated by immunohistochemical and pyrosequencing techniques) and ATRX mutation were recorded where available. The availability of these results varied over the years as new molecular pathology techniques were sequentially introduced into clinical practice [16].

Patients had histopathology subsequently updated as per the WHO 2016 Classification [18]; and included those with recently diagnosed WHO Grade III Pathology or patients with known WHO Grade II Pathology from prior diagnosis but had recent radiological or metabolic progression consistent with anaplastic change. The latter required imaging criteria of progressive MRI T1 radiological abnormality within a six-month period, in addition to either new gadolinium enhancement on MRI or FDG uptake in absence of enhancement [16]. These progressive IDH mutated low grade tumours were presumed to have an equivalent natural history to anaplastic glioma [3] and were included in the study.

### Radiological procedures

Gadolinium-enhanced 3 T MRI was the principal diagnostic procedure at initial diagnosis and RT planning. Tumour was delineated on T1 and T2Flair sequences. Presence of contrast enhancement was recorded as absent, patchy (< 10 mm) or diffuse regions (> 10 mm).

Nuclear medicine imaging with combined FET and FDG-PET was commenced in 2011 and subsequently performed routinely on all patients with IDH mutation. The FET studies were acquired on a Siemens Biograph mCT PET/CT scanner with extended axial field of view and time of flight (ToF) imaging capability; with 20 min dynamic and 10 min static acquisitions following a 3 min FET infusion period [16].

### Radiological classification

An insular tumour for this study was defined as having MRI evidence of infiltration into the gyri of the insular cortex. The insular tumours were then classified radiologically as per the Berger-Sanai Classification [19] with involvement of regions classifying the tumours as occupying Zones I-IV with a further category (Zone V) classifying giant tumours that occupy all zones. This classification reflects the geographical site of tumour within the insular region, related superior and inferior to the line of the Sylvian fissure; and anterior/posterior to a perpendicular plane crossing the foramen of Monro. Patients were described to the zones involved as well as the major zone involved [16].

### Treatment procedures

#### Neurosurgical management

Patients were referred from a number of neurosurgical centres with varying approaches to the extent of surgical intervention and timing of IMRT for anaplastic glioma. Data was recorded regarding the number of salvage craniotomies, the time from initial surgery to radiation therapy and the extent of gross residual disease on MRI T1 and T2Flair sequences prior to IMRT [16].

#### Radiation therapy management

IMRT was utilised for all patients and management was through one clinician. The target volume defined by preoperative and postoperative MRI and importantly any historical MRI imaging, especially for patients with progressive disease after prior surveillance was imported for assistance with target volume determination. FDG-PET and FET-PET were utilised where available for target volume determination [16].

Prior to 2011, the tumour was defined as a gross tumour volume (GTV) for each imaging modality and separate T1, T2Flair, FDG-PET and FET-PET GTVs were delineated. These were combined to produce a final GTV, expanded by 10 mm to a clinical target volume (CTV) and then a further 3 mm to a planning target volume (PTV). This volume received a dose of 59.4Gy in 33 fractions and delivered over a period of 6–7 weeks.

From 2011 with the commencement of routine IDH mutation testing and the recognition of a potential favourable subgroup of patients with long-term survival, a new protocol was commenced for patients with IDH mutation [13]. This incorporated an IMRT integrated boost with two dose levels 59.4Gy and 54Gy defined both by MRI and PET with FDG or FET tracers. The high dose region (GTV59.4) encompassed any areas of gadolinium enhancement, T1 density or FDG uptake. The lower dose region (GTV54) included this volume, as well as region of T2Flair and FET uptake outside of this volume. Patients with wild-type IDH tumours on both immunohistochemistry and subsequent protein sequencing were managed with a single-phase treatment to 59.4Gy as per the EORTC CATNON Protocol [20].

#### Systemic therapy management

The protocol for systemic therapy evolved over the duration of study with the addition of adjuvant temozolomide commencing in early 2012 following release of long-term results of the RTOG and EORTC studies showing improved median survival for patients with oligodendroglial tumours receiving adjuvant chemotherapy [21, 22]. This was extrapolated to the cohort of patients with IDH mutation rather than just those oligodendroglial tumours with 1p19q co-deletion [16].

#### Post-treatment surveillance

Patients were evaluated at 1 month post-IMRT with MRI and then commenced a surveillance programme with 3 monthly MRI for years 1–2, then 4 monthly in year 3 followed by 6 monthly until year 5.

### Study procedures

#### MRI tumour volume calculation

**Fig. 1:**
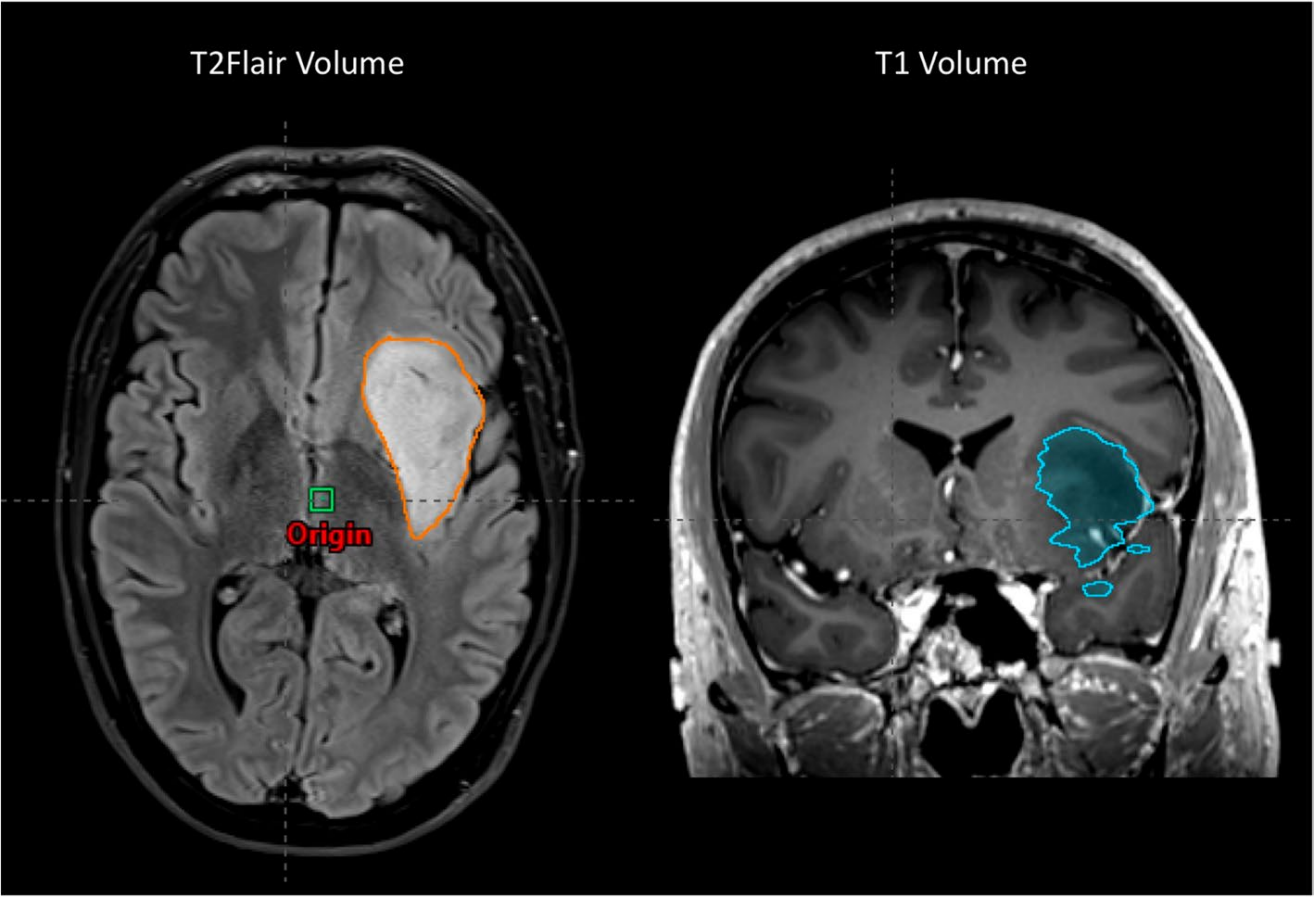
Example of T2Flair and T1 MRI Tumour Volume measurement

Measurements were performed by the lead author, an advanced trainee radiation oncologist. The volume of residual tumour pre-RT was calculated in cm^3^ by contouring the complete volume on MRI fused to the simulation CT scan in the treatment planning system. Surgical cavity was excluded in selection of the maximum dimension in any plane. This was conducted on T1 and T2Flair image sequences (Fig. 1). Any uncertainty over volume delineation was clarified with a neuroradiologist.

**Fig. 2:**
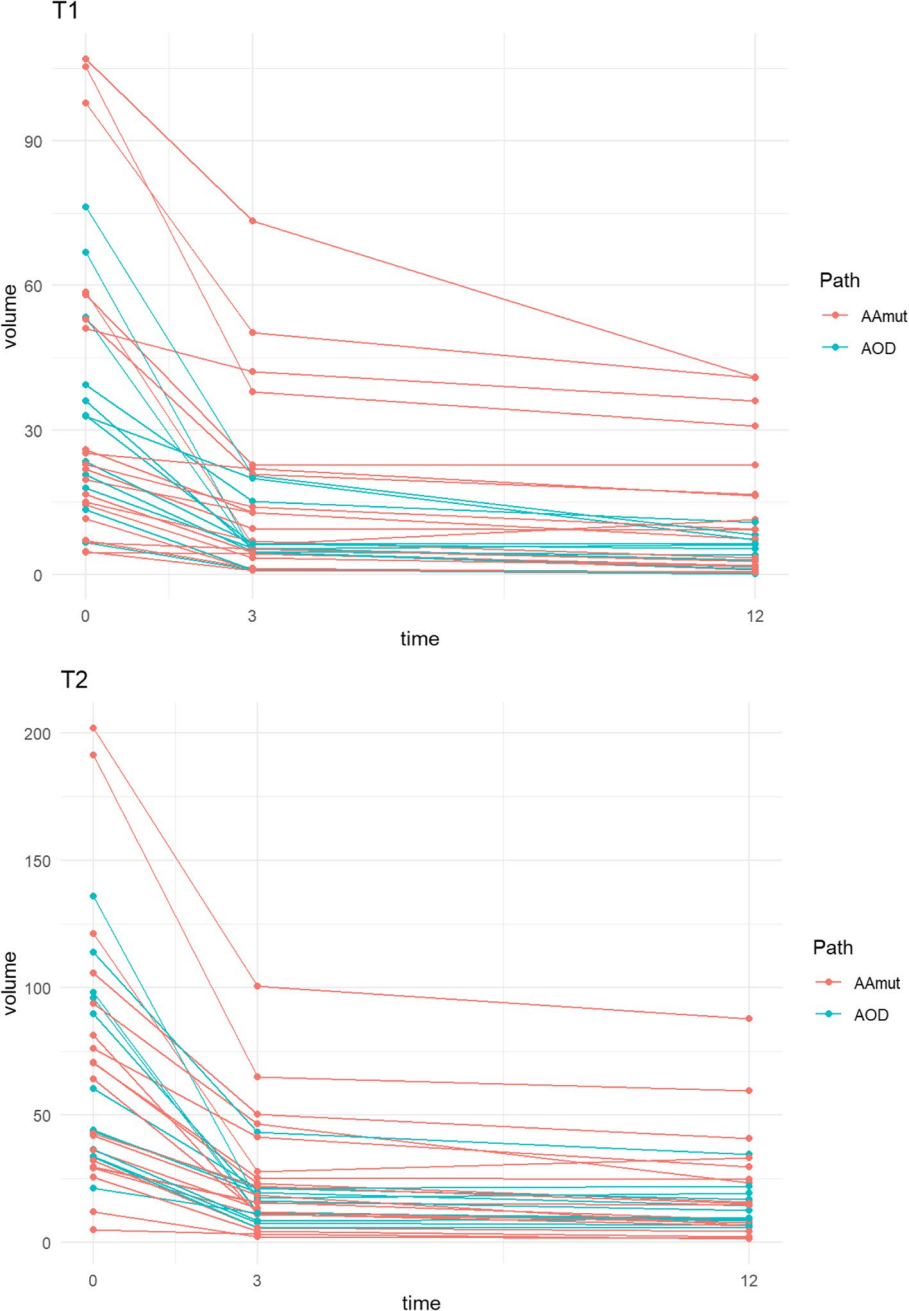
Example of T2Flair and T1 MRI Tumour Volume assessment over timepoints

At set time points for study evaluation, 3 months (month+ 3) and twelve months (month+ 12) following completion of RT, the same procedure was undertaken on these images fused into the radiation therapy treatment planning system (Fig. 2).

Additionally, the presence of contrast enhancement was recorded from T1 with gadolinium sequences and categorised as absent, patchy (< 10 mm) or diffuse regions (> 10 mm). Where measurable, the volume of the largest contrast enhancing area was calculated at each time point [16].

#### Study endpoints

The primary endpoint was change in the MRI tumour volume as measured on both T1 and T2Flair sequences performed at 12 months post-IMRT completion. A secondary endpoint was the similar procedure at 3 months post-RT completion. The volumetric endpoint was calculated as a median reduction for the total group; as well as the median of the volume reductions experienced by each patient.

Relapse-free survival (as measured from date of commencement of RT to date of relapse or last follow-up) was analysed as a measure of treatment efficacy. Relapse was defined using RANO Criteria post 2012 [23]. For patients within 18 months of completion of IMRT, in which uncertainty existed whether the MRI changes were related to tumour or treatment effect, sequential MRIs were utilised to confirm diagnosis but date of relapse was taken from initial MRI if subsequently confirmed. The relapse site was categorised in relation to the IMRT defined GTV (CTV59.4) and defined as: Infield (> 50% within CTV59.4/54); Marginal (> 50% within 20 mm of CTV59.4/54); Distant (> 50% beyond 20 mm from CTV59.4/54); or Combined (synchronous sites of relapse). Additionally, involvement of ventricular system was reported if a distant relapse was evident.

No detailed acute or late toxicity criteria were assessed for this study; however a measurement of longer performance status and impact of therapy were explored using functional outcome measures obtained at month + 12 and month + 60 post-IMRT. These were change in Eastern Co-operative Oncology Group Scale (ECOG) as well as change in Employment Status from baseline.

### Statistical considerations

All patients had clinical and MRI volume data entered on an Excel database at Northern Sydney Cancer Centre and updated for outcome events. Kaplan-Meier estimates of survival distribution were used to calculate relapse-free survival. Volume reductions were calculated at each time point from baseline as both the central tendency measured as median score; and the dispersion of data by the interquartile range (q1–3). Log-rank test was used to investigate associations between the volume reduction and potential predictive factors. All reported *p*-values are two-tailed. Statistical significance was defined as *p* < 0.05 in all cases. IBM SPSS Statistics Version 23 was used for statistical analysis [16].

## Results

**Fig. 3:**
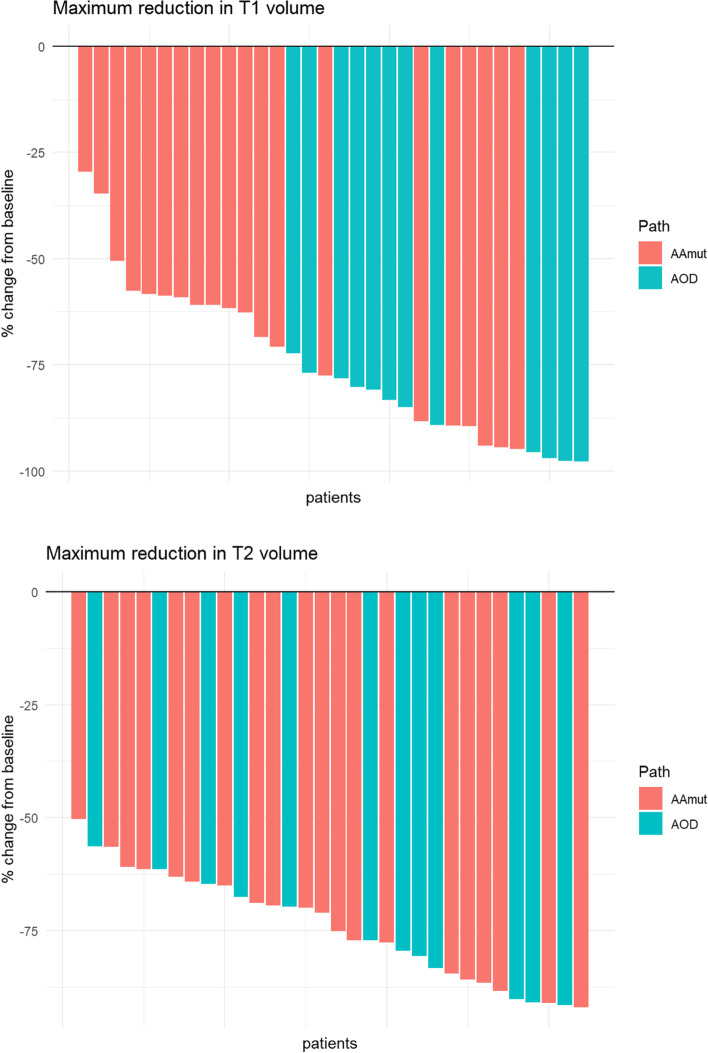
Overall survival for all patients from start of IMRT(n = 32)

One hundred and sixty-one patients were accessed from the Anaplastic Glioma Database who had IDH mutated anaplastic glioma and received management between January 2008 and June 2019. Thirty-two patients had tumours infiltrating into the insular region and were evaluable for the study. At time of data censure in February 2020, five patients are deceased with a 5-year overall survival of 92% (CI 0.81–1.00) (Fig. 3). The median follow-up for survivors is 67.2 months with a range from 6 months to 134.4 months.

Patient characteristics are listed in Table 1. Median age was 38.3 yrs. Forty-seven percent of patients were managed with IMRT at initial diagnosis; and 16% of patients at second or later relapse.

**Table 1:** Patient, Tumour and Treatment Characteristics

	Subgroup	Number (*n* = 32) (%)
Age at Diagnosis	< 40 yrs	17
	> 40 yrs	15
	Median	38 yrs
Number of Craniotomy prior to RT	1	23 (72%)
	2	4 (12%)
	3 or more	5 (16%)
Timing of RT	At initial diagnosis	15 (47%)
	1st relapse	12 (38%)
	2nd relapse	2 (6%)
	Later	3 (9%)
Pathology WHO 2016	AOD	12 (37%)
	AAmut	20 (63%)
Ki67% (preIMRT Surgery) Ki67% (prior Surgery)	Median (*n* = 22)	10.0% (q1–3: 5.0–16.2)
	Median (*n* = 10)	5.0% (q1–3: 2.5–6.5)
ATRX	Retained	16 (50%)
	Lost	8 (25%)
	Unknown	8 (25%)
Pre-Treatment Gad enhancement	Nil	27 (84%)
	< 10 mm	4 (12%)
	> 10 mm / diffuse	1 (3%)
Pre IMRT ECOG performance status	0	13 (41%)
	1	17 (53%)
	2	1 (3%)
	3	1 (3%)
IMRT Technique	Integrated Boost	27 (84%)
	Single	5 (16%)
Temozolomide	Nil	7 (22%)
	Pre- and post-RT	2 (6%)
	Adjuvant post-RT	23 (72%)

All patients had tumours with IDH mutation, and pathologies as per WHO 2016 Classification were AOD in 12 patients and AAmut in 20 patients. Of those AOD patients, 10 patients had molecularly confirmed 1p19q co-deletion and 2 patients managed before 2016 had immunohistochemically features consistent with oligodendroglioma but WHO integrated diagnosis was limited by insufficient tissue. All patients had a histopathological confirmation of anaplastic tumour except for 5 patients; 1 patient had symptomatic tumour growth in less than 6 months, 2 patients had a biopsy only but significant contrast enhancement on MRI with corresponding FDG-avidity on FDG-PET scan, 2 patients had histopathologically confirmed grade 2 tumours with serial surveillance MRI scans showing documented progression to anaplastic tumour with significant contrast enhancement.

IMRT was given immediately after the index surgery in 22 patients, whilst 10 of the 32 patients were managed following progression from a prior known anaplastic (4 patients) or low-grade glioma (6 patients). Of the 22 patients with surgery immediately before IMRT the median ki67% was 10% (q1–3: 5.0–16.2); whilst the 10 patients with no recent surgery the median ki67% was 5% (q1–3: 2.5–6.5).

All patients had radiologically-measurable residual disease on MRI/FET-PET evident prior to IMRT. Twenty-one patients had a maximum diameter of residual tumour exceeding 50 mm; and 16 patients had gadolinium enhancement prior to diagnosis, though only 5 had residual enhancement at time of IMRT. The bulk of disease is reflected by the Berger-Sanai Classification demonstrating 53% of patients had a giant glioma with involvement of all 4 zones. Zone I (anterior and superior) was involved in 84%; though it was only the dominant region in 37% of patients.

All 32 patients received the IMRT to a dose of 59.4Gy as prescribed, and 27 had the integrated boost technique. Sequential temozolomide chemotherapy was delivered in 78% of patients; with 7 patients managed with RT alone prior to the protocol alteration in 2012 when sequential temozolomide was added routinely.

### MRI tumour volume analysis

At baseline the 32 patients had median T1 and T2Flair volumes of 24.3 cm^3^ (range: 4.6–107.0) and 52.2 cm^3^ (range: 5.0–202.0) respectively (Table 2). The 5 patients with contrast enhancement at baseline had minimal enhancement with one patient having 4 cm^3^ but the other four patients were patchy enhancement and less than 0.1cm^3^. Following treatment, all 32 patients were assessable at month+ 3 and 30 patients had month+ 12 assessment.

**Table 2:** Baseline Tumour Volume Parameters pre-IMRT

Endpoint	n = 32 (%)	
2D Residual Tumour (Maximum diameter)		
	< 20 mm	2 (6%)
	20-50 mm	9 (28%)
	> 50 mm	21 (66%)
T1 Volume (Median and range)	24.3 cm^3^	4.6–107.0 cm^3^
T2 Volume (Median and range)	52.2 cm^3^	5.0–201.9 cm^3^
Berger-Sanai Classification Zones (Number and % tumour where zone involved)	I	27 (84%)
	II	20 (62%)
	III	18 (56%)
	IV	26 (81%)
	Giant	17 (53%)
Principal Berger-Sanai Classification Zone (Major Zone Involved)	I	12 (38%)
	II	0 (0%)
	III	3 (9%)
	IV	8 (25%)
	Giant	9 (28%)

**Fig. 4:**
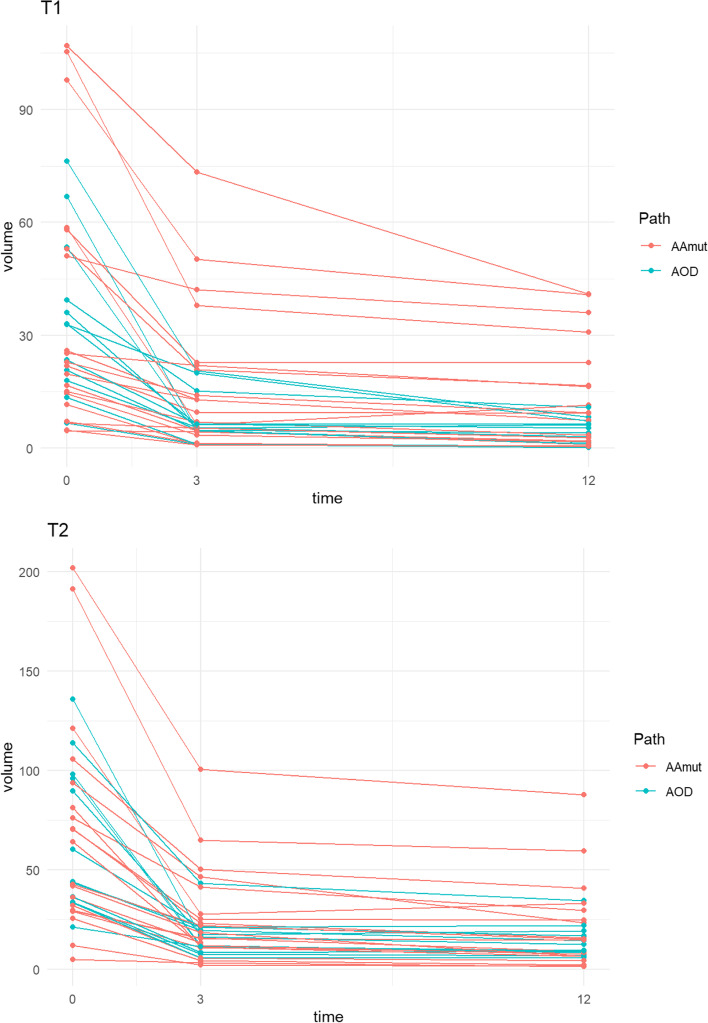
Individual patient absolute MRI Tumour Volume (T1 and T2Flair) reductions at month+ 3 and month+ 12 post-IMRT for pathological subtype

The individual patients’ volume reduction and the rate of change for both T1 and T2Flair volumes at month+ 3 and month+ 12 are demonstrated in Fig. 4. The median T1 volume reduced from 24.3 cm^3^ to 6.3 cm^3^ and 6.2 cm^3^ at months + 3 and + 12 respectively. The median T2Flair volumes paralleled this reduction from 52.2 cm^3^ to 16.1 cm^3^ and 12.4 cm^3^ respectively. At month+ 3, the median volume reduction for T1 and T2Flair was by 67 and 64% respectively; which continued to reduce to 78 and 73% from baseline at month+ 12.

**Fig. 5:**
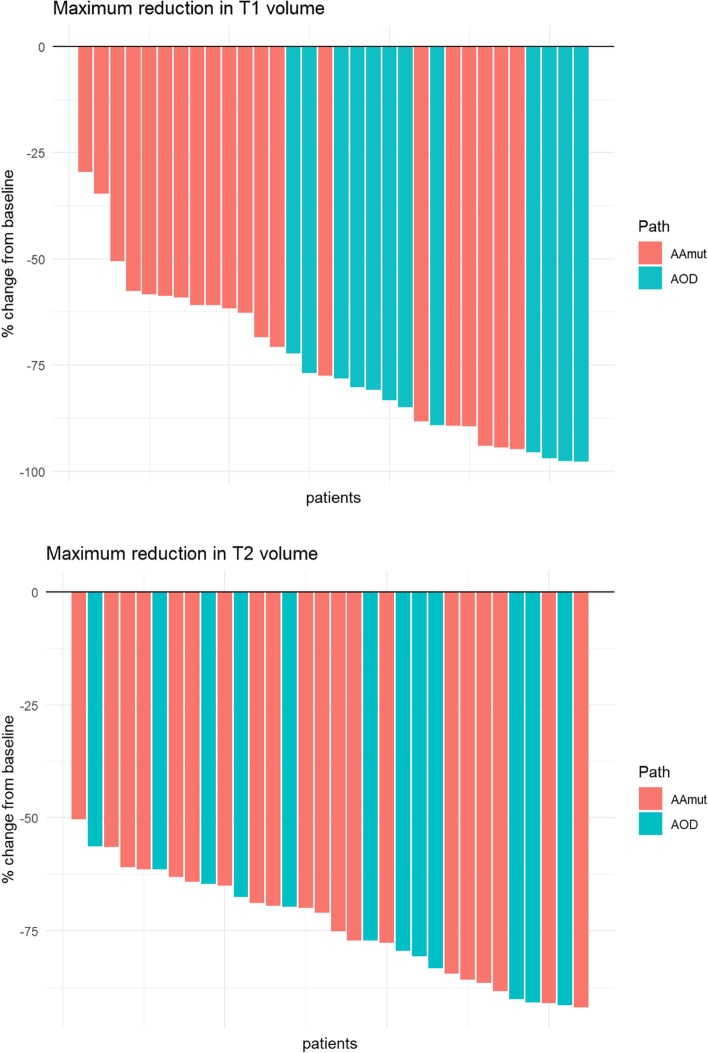
Waterfall plot of individual patient percentage MRI Tumour Volume (T1 and T2Flair) reductions at month+ 12 post-IMRT from baseline for pathological subtype

For individual patients and pathological subtype, the volume reduction at month + 12 are shown as Waterfall Plots (Fig. 5). At month+ 12, 56 and 44% of patients had > 75% volume reduction in T1 and T2Flair volume from baseline.

For the 5 patients with contrast enhancement prior to IMRT, the volume measurements were limited with no consistent pattern of response. At month+ 3, 2 patients had resolution of their enhancement, but a further 1 patient had developed focal enhancement of less than 2.3 cm^3^. The one patient with prior bulky enhancement of 4 cm^3^ had a complete response by month+ 3.

### Predictive factors for MRI tumour volume reduction at month+ 12

Tumour related factors were analysed for association with T1 and T2Flair tumour volume reduction at month+ 12. Only WHO 2016 pathological subtype of AOD compared with AAmut was associated with more T1 volume reduction with median 83 and 63% respectively (*p* < 0.01). This difference was not evident with T2Flair reduction at month+ 12 with median 76 and 71% respectively (*p* = 0.62). No other factors were associated with volume reduction at month+ 12.

### Relationship of progression free survival and MRI tumour volume

**Fig. 6:**
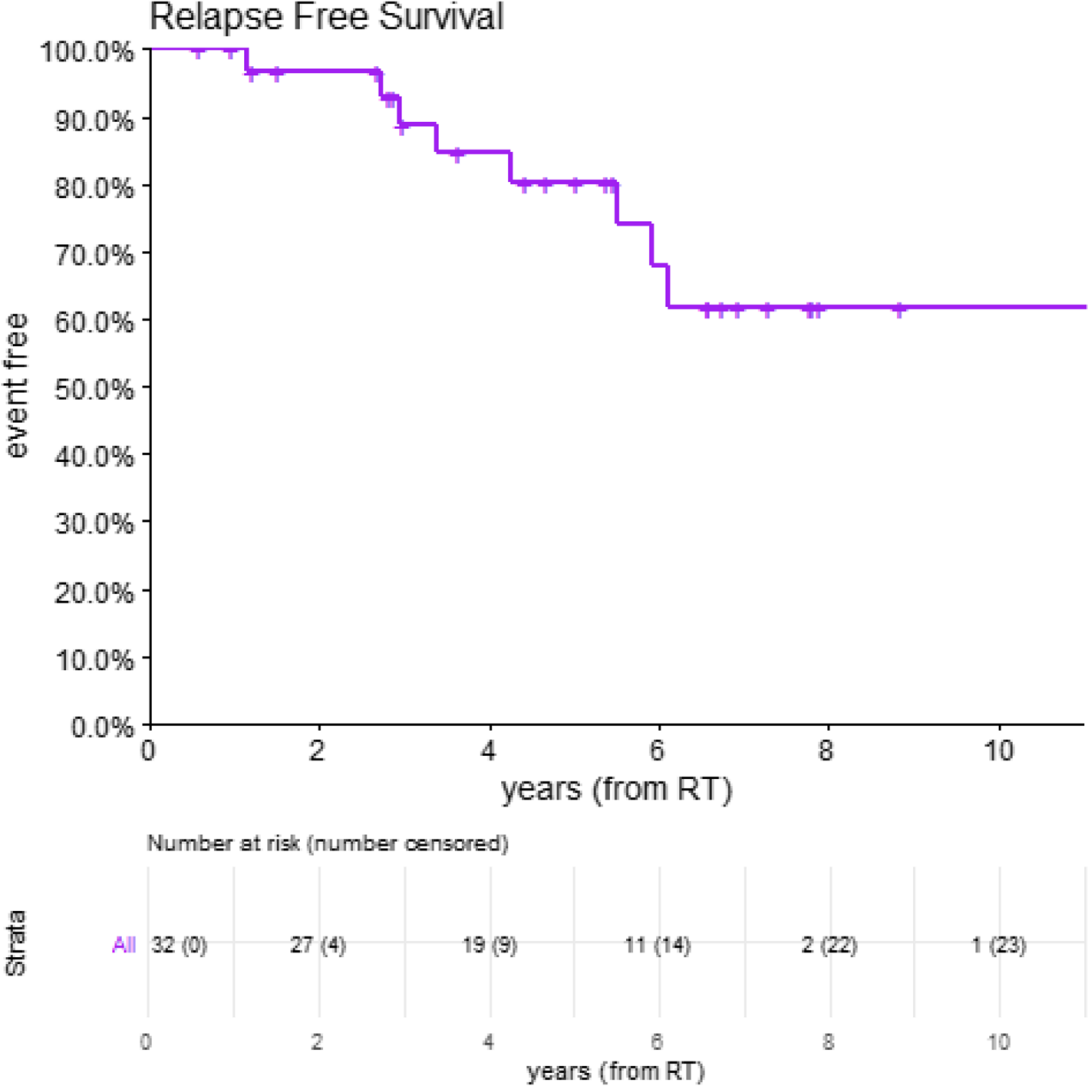
Relapse free survival for all patients from start of IMRT (n = 32)

Nine patients have relapsed with a projected 5-year relapse free survival of 80%; of which eight had an isolated local failure and only one relapse had a component of distant failure (Fig. 6). No factors were associated with relapse, specifically initial T1 volume (*p* = 0.52), initial T2Flair volume (*p* = 0.93), pathological subtype (*p* = 0.12), ki67% (*p* = 0.42), presence of enhancement (*p* = 0.38), Berger-Sanai Classification Giant Glioma (*p* = 0.85) or delayed referral for IMRT (*p* = 0.34). Additionally, the extent of T1 or T2Flair volume reduction at month+ 3 or month+ 12 was not associated with improved progression free survival.

### Functional outcome

At month+ 12 there was an improvement in ECOG compared with baseline with all patients either ECOG 0–1 and the ECOG 0 percentage increased from 40 to 59%. This was similar in month+ 36 with 84% accessible patients being ECOG 0–1. There was one patient with a reduction in ECOG score in the year post-treatment which normalised in the 3-year follow-up period.

In regards to employment, 22 patients could work prior to RT, with 7 patients having major impairment and 3 patients retired. Of these 22 patients working at baseline, 17 were accessible in their third year post IMRT and all remained employed. Five patients of the 17 patients had moved to higher roles, whilst 3 had residual deficits and were employed at a lower level or with restricted duties.

## Discussion

For tumours of the insular region harbouring an IDH mutation there is the potential for long-term survival especially the subgroup with additional 1p19q co-deletion [1–4]. Initial decision-making requires maximising progression free survival without causing treatment related morbidity that could impact on performance status. This study demonstrates excellent tumour volume reduction and progression free survival at early time points following IMRT and temozolomide in a high-risk group of patients with significant residual disease at time of management. Importantly, functional status has been maintained or improved from their pre-treatment level.

Surgical approach in the insular region generally requires a sophisticated neurosurgical approach to minimise the morbidity of resection. The neurosurgical issues that increase intraoperative risk include presence of middle cerebral artery branches (particularly small perforating vessels), involvement of the basal ganglia, adjacent speech connections for the dominant hemisphere and the diffuse nature of these tumours with poor demarcation between tumour and normal brain. Preoperative evaluation may include utilising 2HG spectroscopy to determine the presence of an IDH mutation [24]; functional MRI especially with left sided insular tumours to determine Broca’s area [25]; and PET scans to identify regions of higher grade for targeted resection [26]. Intraoperative guidance utilising awake craniotomy, functional cortical mapping, real-time MRI, endoscopic resection or a combination of these procedures can potentially allow a better understanding of the potential extent of resection [5, 27].

While some studies have demonstrated the survival benefit of maximal debulking for IDH mutated tumours this does remain uncertain in modern times with advanced radiotherapy techniques [11]. Retrospective analyses have attempted to clarify the relationship with residual disease and survival, however interpretation has been difficult given the varied adjuvant therapy policies adopted and the likely differing outcomes from subgroup of AAmut and AOD. The historical data describing a relationship between volume of residual disease and outcome relates to an era prior to molecular classification. The landmark 2008 UCSF research demonstrating the adverse impact of small residual disease volumes on progression-free survival and overall survival predated IDH mutation testing [28]. In this study, there was no uniform policy on role of adjuvant RT, though noting that whilst no patients with complete resection received upfront RT, only 32% of patients with subtotal resection proceeded to RT. For the total group, RT demonstrated a benefit in progression-free survival, however a negative effect on overall survival, presumably related to those patients receiving RT having worse prognostic features. Of note is that insular tumour location was the only brain regional subsite with a significantly worse progression-free and overall survival compared to frontal lobe tumours. This may reflect the need to consider the insular region as a subspecialised site for management protocols.

The most detailed analysis relating to extent of residual disease as a prognostic factor for tumours with IDH mutation is from Rotterdam published in 2018 [29]. This included 28 patients with insular glioma, and that subsite was associated with a higher volume of residual disease after surgery. This study demonstrated a significant association for overall survival with surgery to minimise residual disease for 112 patients with low grade IDH mutated astrocytoma. This existed for all residual volumes from 0.1–5.0 cm^3^ to > 15 cm^3^; though it should be noted that the use of adjuvant radiation therapy was limited to only 49% of patients. This association of improved survival and extent of residual disease was not demonstrated in patients with oligodendroglioma in this study however, authors acknowledge that other studies have shown a surgical benefit for 1p19q co-deleted tumours [30] Additionally, a retrospective review of 12 patients with insular gliomas, showed a median extent of resection of 94% was achievable via a transopercular surgical approach with no reported permanent speech or motor deficits [31].

The authors acknowledge the spectrum of grade 3 tumours that exists within this study. In both AOD and AA, there are patients with predominately grade 2 disease with small foci of grade 3 disease whist other patients with majority higher grade disease. The difference in volume of grade 3 disease may have prognostic implications that have not been addressed in this study and may benefit from separate analysis. In addition to this, anaplastic tumours have traditionally been considered a different cohort to grade 2 tumours and subsequently been treated with different approaches. However, in the era of IDH mutation, the prognostic difference between the two grades is less significant and analysis of a broader cohort could be meaningful.

An approach with IMRT and temozolomide which is aimed at minimising postoperative morbidity should be designed to optimise the therapeutic relationship, with progression-free survival and avoidance of late toxicity. Whilst this study is limited in description of late effects of IMRT, the functional endpoints of excellent performance status and sustained employment suggest that in the median follow-up period of survivors the impact of IMRT is acceptable. This has also been demonstrated in a larger cohort from multiple brain regional sites where functional status in the years post IMRT is high, and most latent impact arises from the immediate postoperative morbidity [13, 32, 33]. While specific metrics on memory and seizure control were not available, the fact that the vast majority of patients were able to return to work with a proportion in higher-functioning roles than pre-radiotherapy, suggests the minimal impact that radiotherapy has on quality of life, in particular with memory issues. It is acknowledged there is a potential for late neurocognitive side effects that may emerge in the 5–10 years after radiotherapy treatment however with improved techniques in radiation treatment these may be less associated with radiotherapy and could be multifactorial including anticonvulsant therapy or relapsed disease [34]. It does demonstrate the importance of continuing long term follow up of clinically relevant functional endpoints.

As knowledge of IDH mutated glioma expands then the potential to predict more adverse tumour subsites may infer a need for a more aggressive initial approach aimed at near-total resection. An expanding knowledge of molecular features, such as CDKN2A heterogenous deletion [35, 36], may then become factors that influence the utilisation of a more aggressive approach with repeat craniotomy seeking maximal tumour reduction prior to delivery of IMRT.

## Conclusion

Large tumour volume reduction of IDHmut AG involving the insular cortex is possible with IMRT and provides excellent tumour control and post-treatment performance status. There was no significant impact of residual post-surgical tumour volume when IMRT was utilised. This approach should be considered as a favourable alternative to potentially morbid debulking surgery for this patient cohort.

## Data Availability

The data generated and analysed during the current study is not publicly available due to patient confidentiality but will be available for sharing after local institutional ethics approval. Contact the corresponding author (Dr Gabrielle Metz) if needed.
